# Metabolic modeling links gut microbiota to metabolic markers of Parkinson’s disease

**DOI:** 10.1080/19490976.2025.2554195

**Published:** 2025-09-25

**Authors:** Tim Hensen, Ines Thiele

**Affiliations:** a Digital Metabolic Twin Centre, University of Galway, Galway, Ireland; b School of Medicine, University of Galway, Galway, Ireland; c School of Microbiology, University of Galway, Galway, Ireland; d Ryan Institute, University of Galway, Galway, Ireland; e APC Microbiome Ireland, Cork, Ireland

**Keywords:** Gut microbiome, metabolic modeling, parkinson’s disease, biomarkers, host-microbiome co-metabolism

## Abstract

Human gut microbiota have been implicated in metabolic disruptions in Parkinson’s disease (PD). However, the underlying mechanisms linking gut microbiota to these disease-related metabolic changes remain largely unknown. In this study, we applied constraint-based metabolic modeling to identify potential causal links between compositional shifts in gut microbiota in PD and metabolic blood markers of PD. We personalized *in silico* whole-body metabolic models with gut metagenomics of 435 PD patients and 219 healthy controls and profiled *in silico* gut microbiome influences on 116 blood metabolites with replicated associations with PD diagnosis. Our analysis identified a reduced capacity of the PD host-microbiome co-metabolism to produce L-leucine and leucylleucine in blood. These metabolic predictions were traced back to lower L-leucine production of *Roseburia intestinalis* and higher L-leucine consumption by *Methanobrevibacter smithii* in PD microbiomes. We further predicted reduced host-microbiome production capacities of butyrate, myristic acid, and pantothenate in the blood of PD patients and linked these associations to reduced relative abundances of *Faecalibacterium prausnitzii*. Finally, lower nicotinic acid production capacities were predicted in PD patients, which were associated with increased relative abundances and increased nicotinic acid consumption of *Ruthenibacterium lactatiformans* in PD. In conclusion, we predicted that the gut microbiome can drive altered blood levels of six metabolites in PD and identified candidate microbial species that may influence these metabolic alterations. These findings may facilitate the development of novel therapies targeting the gut-brain axis in PD.

## GRAPHICAL ABSTRACT

**Figure:**
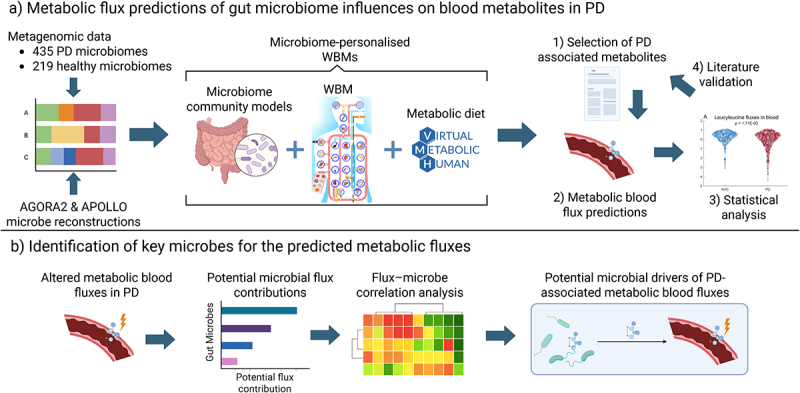

## Introduction

Parkinson’s disease (PD) is a common progressive neurological motor disease, characterized by a loss of dopaminergic neurons, the accumulation of alpha-synuclein proteins, and the formation of neuronal intracellular Lewy body inclusions.^1^ Its pathogenesis is associated with widespread disruptions to physiological functioning and metabolism.^1,2^ Peripheral factors, such as gut microbiota, have been found to contribute to these metabolic perturbations via the production and intestinal uptake of microbial metabolites.^3–5^ PD pathogenesis has been previously linked with several microbe-associated metabolites, including, but not limited to, short-chain fatty acids,^6^ trimethylamines,^7^ polyamines,^8^ and sulfur metabolites.^9–11^ These PD-specific gut microbial contributions to systemic metabolism suggest that gut microbiota could hold promise as a disease marker or even as vectors for novel therapies.^12^ However, the exact microbial role in these PD-associated metabolic perturbations remains poorly understood, as metabolic perturbations are also influenced by a range of further factors, such as environmental exposures, dietary intake, drug usage, and host genetics.^13,14^

Despite this complexity, metagenomic and metabolomic correlation methods have successfully identified PD-associated microbe-metabolite pairs.^15,16^ Nevertheless, mechanistically linking gut microbiota to perturbed metabolites in PD patients remains difficult using purely correlation-based methods. Therefore, computational modeling approaches have been used to complement correlation-based methods by providing mechanism-based predictions on microbe-metabolite associations.^17^ The constraint-based reconstruction and analysis (COBRA) framework^18^ has established itself as a powerful method for linking gut microbiota with disease-related metabolic shifts.^19,20^

The COBRA framework has found particular success in finding microbe-metabolite associations in human health and disease through the creation and analysis of personalized microbiome community models.^9,10,19,21,22^ The creation of these microbiome community models was enabled by large-scale resources of genome-scale reconstructions for human microbes, such as AGORA (818 microbes),^21,23^ AGORA2 (7,302 microbes),^24^ and APOLLO (247,092 microbes),^25^ which enabled metagenomic reads to be mapped onto genome-scale reconstructions of corresponding microbes.

Previously, microbiome community modeling in PD patients identified alterations in microbiome sulfur metabolism,^9^ which were mechanistically linked to increased relative abundances of *Akkermansia muciniphila* and *Bilophila wadsworthia* in PD microbiomes. A follow-up study confirmed the associations with sulfur metabolism but also identified increased gut microbiome production capacities of methionine and cysteinylglycine in PD microbiomes and a positive association between microbial pantothenate production capacity and non-motor PD symptoms.^10^ Crucially, these previous modeling studies did not account for the effects of metabolic host-gut crosstalk or investigate gut microbial influences on downstream host-side metabolites. However, investigations of host-gut crosstalk have become possible since the introduction of sex-specific, organ-resolved whole-body metabolic models^26^ (WBMs). Gut microbiome-personalized WBMs have already contributed to novel links between host-microbiome co-metabolism and Alzheimer’s disease.^27,28^

Here, we followed up on previous modeling efforts on PD microbiomes by investigating host-microbiome co-metabolism using gut microbiome-personalized WBMs. Our analysis revealed reduced metabolic contributions of PD microbiomes to blood levels of L-leucine, leucylleucine, butyrate, nicotinic acid, pantothenate, and myristic acid. We associated these predicted metabolic alterations with microbial shifts in PD, identifying *Roseburia intestinalis* and *Methanobrevibacter smithii* as potential key influencers of the predicted reduced L-leucine and leucylleucine microbiome contributions in PD. *Faecalibacterium prausnitzii* was identified as a candidate driver of the predicted changes in butyrate, pantothenate, and myristic acid blood levels, while *Ruthenibacterium lactatiformans* was identified as a key microbe for the lower predicted microbial contributions to nicotinic acid in blood. Taken together, this study links blood metabolic markers of PD with the gut microbiome through the application of large-scale metabolic models of host-microbiome co-metabolism.

## Results

**Figure 1.**
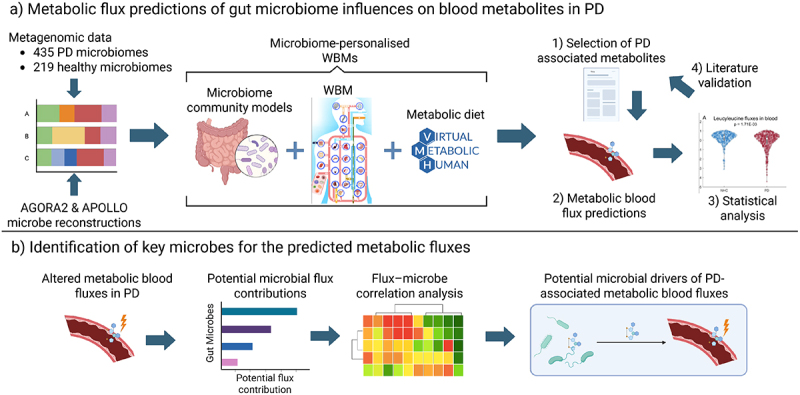
Schematic overview of the study design created with BioRender. a) Gut metagenomic reads were mapped onto the microbial metabolic reconstructions in the AGORA2 and APOLLO resources. The corresponding microbial reconstructions were then combined into microbiome community models and personalized with the species-level relative read abundances. The personalized microbiome community models were joined with male and female WBMs corresponding to the sex of the microbiome sample donor and were given an average Western metabolic diet. Metabolic blood fluxes were predicted for 116 metabolites with previously reported metabolomic PD associations and were statistically analyzed to find metabolites whose alterations may be driven by compositional changes in PD microbiomes. b) For the metabolites with altered metabolic blood flux predictions, we identified metabolite-associated subsets of the gut microbiomes by calculating which microbial species in the WBMs could have contributed to the changed predicted metabolic fluxes. The species relative abundances of these microbial subsets were then correlated with their corresponding metabolic flux predictions to identify potential key microbes for metabolites with altered metabolite production potentials in PD patients.

In this study, we aimed to identify altered blood metabolites in PD patients, whose alterations may be driven by compositional changes in PD microbiomes. Therefore, we systematically investigated the capacity of the microbiome-personalized WBMs to produce PD-associated metabolites by predicting the maximal flux of metabolite accumulation in blood. We analyzed 116 meta-analyzed blood metabolites with replicated associations to PD diagnosis in a cross-sectional cohort of microbiome-personalized WBMs generated from 435 PD gut microbiomes and 219 gut microbiomes of neurologically healthy controls (Figure 1(a), Methods). The predicted metabolic fluxes were then associated with PD diagnosis to identify metabolites with altered metabolic flux capacities in WBMs, which were personalized with PD microbiomes. These metabolites were validated against 74 meta-analyzed studies that reported metabolomic associations with PD. As the analyzed WBMs were only personalized with gut microbiomes, the predicted changes in metabolic fluxes could be traced back to compositional differences in the gut microbiomes. This approach allowed us to identify the key microbial drivers of the predicted metabolites with altered blood fluxes from PD microbiomes (Figure 1(b), Methods). We leveraged the deterministic structure of the metabolic models to identify which microbial species could have contributed to the predicted metabolic fluxes and calculated how much each microbial species could have contributed to the predicted blood fluxes. These potential microbial contributions to the predicted metabolic fluxes were then used to identify subsets of potentially influential microbial species for the metabolites with PD-associated metabolic flux predictions. Next, we correlated the relative abundances of each metabolite-associated microbial species with their corresponding metabolic fluxes to identify key microbial contributors to the blood metabolites, for which PD-associated metabolic blood fluxes were predicted. Finally, we validated the identified microbe-metabolite associations in PD by assessing if the identified microbial species were previously reported to associate with PD in the same metagenomic samples.

### Study cohort

For this study, we re-analyzed a published gut metagenomics dataset^29^ from a cross-sectional cohort of 490 PD patients and 234 neurologically healthy controls living in the city and surrounding region of Birmingham in Alabama, USA. Before analyzing the cohort, five samples were removed from the dataset, as these samples were collected via a swab instead of the OMNIgene GUT Collection kit. In addition, 65 samples were removed due to missing sample metadata in one or more variables (Methods). After removing these samples, 654 samples were used for analysis, including 435 PD patients and 219 neurologically healthy controls (Table 1). The PD patients had a mean age of 68.02 (SD = 8.60), while the controls were slightly younger (Mean = 65.01 y, SD = 8.93). Men were also overrepresented among PD patients, with a male-to-female ratio of 1.87, which was slightly higher than previously reported^30^ average incidence ratios. In contrast, women were overrepresented in the neurologically healthy control group, with a male-to-female ratio of 0.69. The cohort also included metadata on co-morbidities, lifestyle, and medication usage, which have been previously discussed by Wallen et al.^29^ (2022). The population characteristics for the filtered samples were similar to the unfiltered dataset (Table 1).

**Table 1.** Summarized characteristics of the study cohort. This metadata table is adapted from Wallen et al. (2022) and includes recalculated statistics for the analyzed samples for the sample metadata used in this study. In total, 654 gut microbiome samples were analyzed, of which 435 were taken from PD patients and 219 were taken from controls. For each metadata variable, the number of samples (N) is shown for which information was available in PD patients and the controls. The columns for the summary statistics show the mean average and standard deviation (SD) of age in years and body mass index (BMI) in kg/m^2^ for PD patients and controls. For the other variables, binary outcomes (yes/no) and their corresponding percentages are shown. P-values for differences in age and BMI were obtained from a two-sample Wilcoxon rank-sum test. A Fisher’s exact test was used to calculate p-values, odds ratios (OR), and 95% confidence intervals (CI) for the other variables.

Metadata	N	PD patients	N	Controls	P-value	OR [95% CI]
Male samples (% male)	435	274 (62.99)	219	65 (29.68)	7.67E–16	0.25 [0.17—0.35]
Mean age in years ± SD	435	68.48 ± 8.57	219	65.79 ± 8.78	2.07E–04	
Mean BMI in kg/m^2^ ± SD	435	27.99 ± 5.47	217	28.52 ± 6.48	8.06E–01	
Alcohol consumption	435	160 (36.78)	219	112 (51.14)	5.55E–04	1.8 [1.29—2.5]
Laxatives	435	131 (30.11)	219	23 (10.50)	6.04E–09	3.67 [2.28—5.92]
Pain medications	435	97 (22.3)	219	36 (16.44)	8.12E–02	1.46 [0.96—2.23]
Depression, anxiety, and mood medications	435	165 (37.93)	219	50 (22.83)	1.00E–04	2.07 [1.43—2.99]
Antihistamines	435	70 (16.09)	219	70 (31.96)	4.91E–06	0.41 [0.28—0.6]
Sleep aid	435	175 (40.23)	219	58 (26.48)	5.33E–04	1.87 [1.31—2.67]

### Generation of gut microbiome-personalized WBMs

Next, we generated microbiome-personalized WBMs from the gut metagenomics data. First, the taxonomic species in the microbiome data were mapped onto the AGORA2^24^ and APOLLO resources,^25^ which included, respectively, 7,302 and 247,092 genome-scale metabolic reconstructions of microbial genomes. A total of 461 microbial species could be mapped out of a total of 672 microbial species in the gut microbiomes (Table 2, Table S1). The mapped 461 microbial species accounted for an average of 93% of the pre-mapped read counts. Species richness in the mapped microbiomes was decreased from an average of 87.27 (standard deviation, SD = 17.73) to 72.44 (SD = 14.53) microbial species. However, the phylum-level relative abundances remained largely stable after the mapping procedure (Table S1). Relative abundances for *Firmicutes* and *Bacteroidetes*, the most abundant phyla in the microbiomes, changed from 41.96% to 41.97% and from 40.38% to 40.39%, respectively. All unmapped microbial species were removed from the microbiomes for the remainder of the analyses. Furthermore, we filtered the mapped microbiomes on microbial species with a relative abundance of at least 0.0001% to remove potential technical artifacts from the PCR, sequencing, or metagenomic processing steps. This step resulted in the removal of 19 additional microbial species. The remaining 442 mapped microbial species were then used to generate personalized metabolic microbiome models. These microbiome models were created from species-level metabolic reconstructions from AGORA2^24^ and APOLLO^25^ and joined together in a shared microbiota lumen per the species presence data in each sample. The species’ relative abundances were integrated into the microbiome models through the addition of a community biomass reaction, which ensured proportional growth and metabolic contributions of the mapped microbial species (Methods). To account for host metabolism, the microbiome models were coupled with the large intestinal lumen of sex-matched male and female WBMs (Methods). The resulting microbiome-personalized WBMs (Table S2) were then parameterized with a daily dietary intake (Table S3). As no information on diets was available, all models were given the average Western diet^31^ (Methods). In summary, we generated microbiome-personalized sex-specific WBMs from gut metagenomics data for a cohort of 654 individuals. Overall, 93% of the gut metagenomic reads could be mapped onto the AGORA2 and APOLLO resources and presented in the WBMs.

**Table 2.** Gut metagenomic reads available for analysis before and after taxonomic mapping of metagenomic species on microbial species in the AGORA2 and APOLLO resources.

Statistic	Before mapping	After mapping	Fraction of reads covered
Total number of microbial species	672	461	69%
Mean species richness (SD)	87.27 (17.73)	72.44 (14.53)	83%
Mean read counts (SD)	19,087,685 (9,949,944)	17,744,869 (9,392,650)	93%

### Selection of PD-associated metabolites for analysis

To ensure that all modeling predictions were relevant to PD, we assessed only metabolites in the microbiome-personalized WBMs with previously known, replicated associations with PD. Therefore, we used a meta-analysis of 74 metabolomic studies on PD patients,^32^ which identified 190 metabolites to be associated with PD diagnosis in more than one study. Of these 190 metabolites, 116 were present in the blood compartment of the WBMs and belonged to 9 biochemical superclasses, 24 classes, and 34 subclasses (Table S4).^32^ Organic acids (46 metabolites) were the most abundant superclass, followed by lipids (26 metabolites). Within the organic acids, carboxylic acids, which include amino acids and peptides, were the largest biochemical class with 39 metabolites. The selected lipid metabolites contained sixteen fatty acyls, which include fatty acids, and six steroid metabolites, including five bile acids. The analyzed metabolites were further involved in 42 metabolic subsystems, including alanine and aspartate metabolism (11 metabolites) and fatty acid oxidation (10 metabolites) were the most common metabolic subsystems. The associated metabolic subsystems captured 26% of all 161 metabolic subsystems in the VMH (virtual metabolic human) database,^31^ indicating that the curated metabolites capture a diverse range of metabolic pathways. Not all selected metabolites were present in the gut microbiota lumen. Only 72 metabolites could be produced in microbial pathways (Table S4). The remaining 44 metabolites, however, could be indirectly influenced by their metabolic precursors and could thus still be influenced by gut microbiota. Taken together, we systematically selected 116 metabolites for further analysis in the WBM blood compartments, all of which had known replicated metabolomic associations with PD diagnosis. Although this set of metabolites likely represents a fraction of all PD-associated metabolites, it captures major parts of PD-associated metabolism, containing 61% of the known metabolites with replicated metabolomic PD associations, nine biochemical superclasses, and is involved in 26% of all metabolic subsystems in the VMH resource.

### Assessment of PD-specific metabolic shifts in predicted host-microbiome fluxes

**Figure 2.**
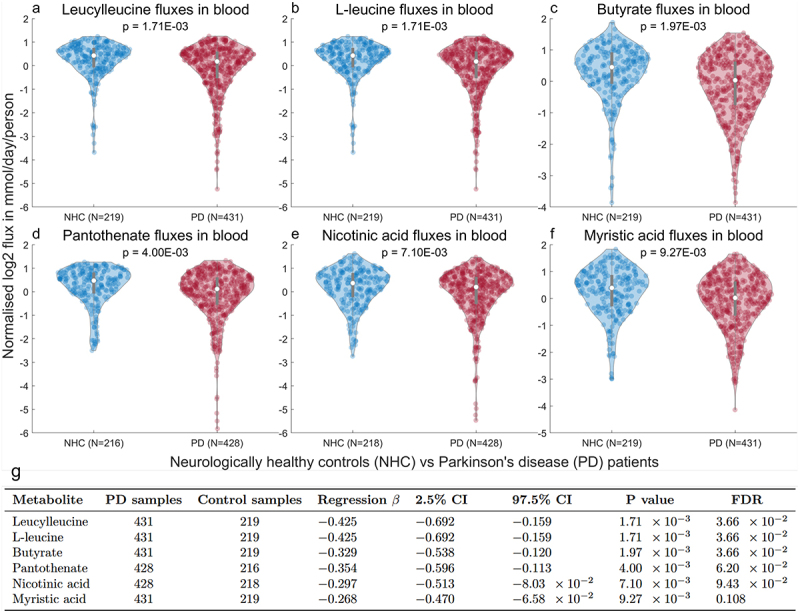
Predicted host-microbiome metabolite production potentials in blood of PD patients and neurologically healthy controls. (a-f) Predicted fluxes in PD patients (red) and neurologically healthy controls (blue) for selected metabolites with the strongest associations with PD status. The shown predicted fluxes in mmol/day/person are log2 transformed and normalized via z-transformation. Identical associations with PD status are shown for leucylleucine and L-leucine, indicating that the predicted gut microbiome influences on L-leucine and leucylleucine fluxes in blood occur through a shared metabolic pathway. G. Table summarizing the logistic regression results of the selected metabolites in a-f. The number of samples for which fluxes could be predicted is shown for PD patients and controls. The regression β shows the log odds ratios for PD status against the predicted fluxes. The negative values β indicate that the predicted blood flux potentials of PD patients are associated with lower predicted fluxes compared to WBMs of controls. The 2.5% and 95% CI further represent the 95% confidence intervals.

Next, potential gut microbial influences were investigated in the microbiome-personalized WBMs. Using flux balance analysis (FBA),^33^ we predicted maximal metabolic fluxes in blood (Methods) in the WBMs. After obtaining the predicted fluxes, 23 metabolites were removed because they were not influenced by the gut microbiomes in 95% of samples or more, resulting in 93 analyzed metabolites. Next, the flux predictions were inspected for outlier samples (Methods), and three samples from PD patients were removed. To assess whether the predicted gut microbiome contributions to the blood metabolites associated with disease status, we performed multiple logistic regressions on PD status against the predicted fluxes, while controlling for relevant confounding factors (Methods). After p-value correction for the false-discovery rate (FDR, Methods), five metabolites were identified that were potentially associated with PD status (FDR <0.1, Figure 2, Table S5). In addition, myristic acid (FDR = 0.108, *p* = 0.009) was also included in further analysis, despite narrowly missing the set cutoff. The top hits in this analysis were L-leucine, leucylleucine, butyrate, pantothenate (vitamin B5), nicotinic acid (vitamin B3), and myristic acid. These hits were all found to have lower predicted fluxes in PD patients compared to controls (Figure 2, Table S5). Notably, the predicted associations with PD status were nearly identical between L-leucine and leucylleucine (Figure 2a). This prediction suggests that gut microbial influences on blood L-leucine and leucylleucine occur through a shared metabolic pathway. Finally, we evaluated whether the associations between the predicted blood fluxes and PD status differed between male and female samples or were influenced by age (Methods). Therefore, two further logistic regression analyses were performed with interaction terms for 1. flux and sex interaction and for 2. flux and age interaction (Methods). These regressions, however, could not find sex-specific or age-related associations for the six PD-associated metabolic fluxes (Table S5), suggesting that our results were not dependent on sex or age. To summarize, we associated flux predictions of 93 metabolites with PD status and found lower flux predictions in PD patients for six metabolites.

### Systematic validation of microbiome-personalized WBM predictions

To validate the lower blood flux predictions in the microbiome-personalized WBMs of PD patients, we consulted the meta-analysis by Luo et al. (2024)^32^ and qualitatively compared our modeling predictions with previous metabolomic findings from 15 metabolomic studies that passed the quality checks (Methods) in the meta-analysis.^32^ Our predicted gut microbiome influences aligned completely with previous metabolomic findings for pantothenate^15,34,43–45^ and leucylleucine^34,35^ (Table 3). In contrast, the predicted lower microbiome-influenced production of myristic acid has not been previously found in the consulted metabolomic studies.^9,38,46^ For the remaining metabolites, no strict consensus could be found between the consulted metabolomic studies. However, the predicted lower blood L-leucine fluxes aligned with all consulted studies on serum^35,36^ and plasma^9^ metabolomic samples. But the predictions contradicted findings from a study on urine samples^38^ and a study, which found higher L-leucine levels in both fecal and plasma metabolomics^15^ of PD patients. Furthermore, our butyrate flux predictions aligned with findings from two studies on fecal metabolomic samples^39,40^ and a study on whole-blood metabolomics,^41^ while contradicting previous findings on plasma^39^ and urine^42^ samples. Finally, the nicotinic acid blood predictions matched with previous findings on fecal^15^ and whole blood samples,^41^ while differing from a previous finding on urine samples.^38^. Taken together, our modeling predictions were largely confirmed by previously analyzed metabolomic associations with PD status, except for our predictions on myristic acid.

**Table 3.** Comparisons of the predicted gut microbiome influences on blood metabolite productions with previous metabolomic findings from meta-analyzed studies analyzed from Luo et al. (2024).^32^

Metabolite	VMHID	Meta-analyzed studies that validate the modeling predictions in:	Meta-analyzed studies that do not validate the modeling predictions in:
Leucylleucine	leuleu	Serum^34,35^	
L-leucine	leu_L	Serum,^15,35,36^ Plasma^9,15,37^	Urine,^38^ Feces^15^
Butyrate	but	Feces,^39,40^ whole blood^41^	Urine,^42^ plasma^39^
Nicotinic acid	nac	Feces,^15^ whole blood^41^	Urine^38^
Pantothenate	pnto_R	Plasma,^43^ serum,^34,44^ feces,^15^ brain tissue^45^	
Myristic acid	ttdca		Plasma,^9,46^ Urine^38^

### Identifying potential gut microbial contributors to the predicted blood fluxes

Next, we interrogated the FBA solutions to identify which microbial species were bottlenecks in the flux predictions. We extracted the flux-associated shadow prices of microbial biomass compounds for each microbial species (Methods). The shadow price is a routinely used sensitivity metric in COBRA modeling that measures the linear change in the predicted flux as the result of a change in metabolite availability.^47^ By checking if a non-zero shadow price was found for a change in abundance of a pan-species biomass metabolite, the influence of microbial growth on the predicted metabolite flux could be identified. Out of the 442 microbial species in the microbiome-personalized WBMs, 435 species were found to potentially contribute to L-leucine and leucylleucine fluxes in blood in one or more samples (Table 4, Figure S1). Butyrate had 90 potential microbial contributors, while for pantothenate, nicotinic acid, and myristic acid, respectively, 341, 433, and 187 microbial contributors were identified.

**Table 4.** Summary statistics on the number of microbial species that limited the blood flux predictions for the selected metabolites. Columns two to four show the total number of potential microbial contributors, the mean average, and the standard deviation of microbial contributors across the cohort. Columns five to seven show the same summary statistics for a filtered set of analyzed microbial contributors to the predicted fluxes.

	Identified microbial contributors			Analyzed microbial contributors		
Metabolite	Total	Mean	SD	Total	Mean	SD
Leucylleucine	435	70.72	14.35	62	35.38	7.88
L-leucine	435	70.74	14.35	62	35.18	7.80
Butyrate	90	19.38	4.97	18	12.35	3.17
Pantothenate	341	57.54	12.71	45	28.54	7.07
Nicotinic acid	433	69.37	15.27	57	34.51	8.11
Myristic acid	187	33.11	10.38	33	20.75	4.32

To identify which of these gut microbial influencers of the flux predictions could potentially explain the lower predicted blood fluxes in PD models, we extracted the potential microbial species flux contributions from the FBA predictions (Methods, Table S6), which were defined as the maximal change in predicted flux observed when a microbiome-personalized WBM was restored to its original state after the removal of a microbial species. All microbial species were then ranked based on their average metabolic contribution potentials to the predicted metabolic blood fluxes of the selected metabolites and pruned to find the smallest set of microbial species that could contribute to, on average, 95% of the total potential gut microbiome flux contributions (Methods, Table S6). The resulting pruned sets of flux-associated microbial species were much smaller than the original sets (Table 4), showing that most of the predicted gut microbiome influences were the result of a minority of the gut microbial species in the models.

### Bacteroides uniformis *and* Bacteroides vulgatus *were the top contributors to PD-associated fluxes, but did not link with PD status*

**Figure 3.**
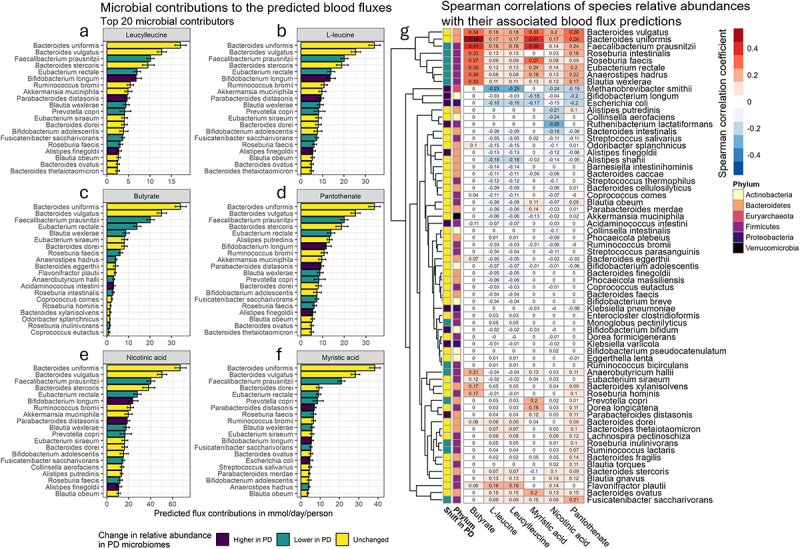
(a-f). Sensitivity of the predicted fluxes toward removing a microbial species. The bar plots show the mean average reduction in predicted fluxes (x-axis) upon removing a microbial species (y-axis). The error bars indicate the 95% confidence intervals for the means. The confidence intervals and the mean averages were calculated from a population of 50,000 bootstrapped samples. The color annotations represent the previously found^29^ shifts in relative abundances in the analyzed PD microbiomes. Blue bars indicate microbial species for which no shift in PD microbiomes has been detected, while red bars represent the microbial species with decreased relative abundances. Lastly, the microbial species with higher relative abundances in PD microbiomes are represented by the green bars. The largest reductions in fluxes were found when removing *B. uniformis*, *B. vulgatus*, and *F. prausnitzii* for all shown metabolites. However, only *F. prausnitzii* has been previously found to associate with PD status. The bar plots show only the top 20 most flux-sensitive microbial species. (g). Spearman correlations between predicted metabolic fluxes in blood and relative microbial species abundances. Correlations are shown for six selected metabolites with lower predicted fluxes in PD patients and microbial species that contributed to the predicted fluxes. Correlation coefficients of zero represent microbial species that could not contribute to the predicted blood fluxes of the associated metabolites. Shown are the sets of microbial species that could together contribute to, on average, 95% of the total potential gut microbiome contributions to their corresponding metabolite blood fluxes.

Next, we assessed which microbial species were the most important contributors to the predicted fluxes of the selected metabolites. The microbial species were first ranked on their average contribution potential to the predicted fluxes. These potentially contributing microbial species were then correlated with the flux predictions and annotated with their previously found species-level shifts in PD microbiomes from the same study population^29^ (Methods). We identified *Bacteroides uniformis* and *Bacteroides vulgatus* as the microbes with the highest potential metabolic contributions to all six differentially produced metabolites in blood (Figure 3, Table S6). This finding showed that both gut microbes could be major contributors to blood levels of these metabolites via the production of the analyzed metabolites or their precursors. The larger metabolic contribution potential was explained by the relative abundances of these microbial species, as they were also the top two most abundant microbes among the gut microbes that were included in the models. The relative abundances of *B. uniformis* and *B. vulgatus* correlated well with the predicted fluxes, especially with butyrate (Figure 3(g), Table S7), which was the only analyzed metabolite that could not be produced by the host and was not present in the diet (Table S4). However, the relative abundances of *B. uniformis* and *B. vulgatus* have previously not been associated with PD status for the analyzed samples^29^ (Figure 3, Table S8), suggesting that these gut microbes might not explain the lower flux predictions in PD models. Despite *B. uniformis* and *B. vulgatus* being potential major producers of the analyzed microbial metabolites, their lack of association with PD status could suggest a more complex role of these gut microbes in PD-associated perturbations in blood for the tested metabolites.

### *Lower relative abundances of* F.prausnitzii *in PD microbiomes linked with lower predicted production of butyrate and myristic acid in blood*

Besides *B. uniformis* and *B. vulgatus*, our results also identified large metabolic contribution potentials for all six metabolites for *F. prausnitzii*, *Eubacterium rectale*, and *Blautia wexlerae*. Their potential metabolic contributions could again be attributed to their high relative abundances, which were the third highest (Table S1) of the mapped microbial species. In contrast to *B. uniformis* and *B. vulgatus*, these gut microbes had lower relative abundances in PD microbiomes compared to gut microbiomes of neurologically healthy individuals, meaning that their lower relative abundance in PD patients could have influenced the flux predictions. The correlation analysis further found moderate correlations with predicted fluxes for butyrate, myristic acid, and pantothenate (Figure 3(g)). The strongest correlations were found for *F. prausnitzii* with both butyrate and myristic acid blood fluxes. In contrast, weak correlations were found for L-leucine, leucylleucine, and nicotinic acid across the three microbial species (Figure 3(g)). These weaker correlations could be explained by the larger number of microbial species that contribute to L-leucine, leucylleucine, and nicotinic acid, diluting the relative metabolic influences on the predicted fluxes. Taken together, we found that lower abundances of *F. prausnitzii*, and to a lesser extent, *E. rectale* and *B. wexlerae*, potentially contribute to lower microbial production of blood metabolites in PD patients.

### *PD-associated increased* Bifidobacterium longum *relative abundances correlated with lower predicted branch-chained amino acid and vitamin B blood fluxes in PD patients*

We also identified microbial contributors to the fluxes with higher relative abundances in PD patients. One of these contributors was *Bifidobacterium longum*, which had large metabolic contribution potentials for L-leucine, leucylleucine, pantothenate, and nicotinic acid (Figure 3(a,b,d,e)). When correlating the *B. longum* relative abundances with flux predictions for these metabolites, negative correlations were found (Figure 3(g)). This negative correlation indicates that a higher relative abundance of *B. longum* associated with lower gut microbiome contributions to L-leucine, leucylleucine, pantothenate, and nicotinic acid, and can be interpreted as *B. longum* potentially limiting other microbial producers of these metabolites. Another major microbial contributor to the predicted fluxes of the selected metabolites with higher relative abundances in PD microbiomes was *Parabacteroides distasonis*. In contrast to *B. longum*, *P. distasonis* had weakly positive correlations with its associated predicted metabolic fluxes (Figure 3(g)). This result could indicate that *P. distasonis* was less important than *B. longum* for the predicted reduction in the blood fluxes from controls to PD patients.

### *Higher abundances of* Escherichia coli *and* M. smithii *in PD microbiomes linked with lower production of PD-associated metabolites*

Other potential microbial influencers of the predicted fluxes were *Escherichia coli* and *M. smithii*. Despite these microbial species not being among the top microbial contributors to the predicted fluxes, they were top negative microbial correlates with the selected metabolites, except for butyrate (Figure 3(g)). In particular, high negative correlations were identified for *M. smithii* relative abundances and predicted fluxes for L-leucine and leucylleucine. When cross-matching this finding with previously found associations with PD diagnosis, we found that the relative abundances of both *E. coli* and *M. smithii* positively associate with PD status in the analyzed samples. This finding suggests that elevated relative abundances of *E. coli* and *M. smithii* in PD microbiomes could contribute to lower predicted levels of microbial metabolites in blood.

### Combinatorial optimization identified microbial clusters that potentially explain the predicted flux associations with PD status

**Figure 4.**
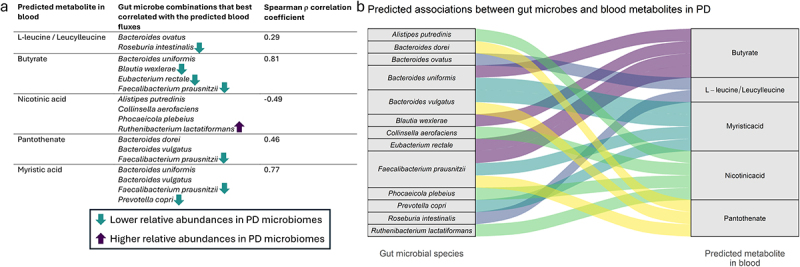
Microbial combinations of metabolic contributors to the predicted blood fluxes that best correlated with the predicted fluxes. Shown are only the largest sets of microbial combinations that improved the Spearman correlation coefficients with the predicted blood fluxes by |ρ|>0.05 compared to any smaller combination of microbial species. *a*. shows the microbial species that together best predicted the blood fluxes, the associated Spearman correlation, and the shift in relative abundances in PD microbiomes. The microbial combinations for butyrate and myristic acid strongly correlated with their associated blood fluxes, meaning that most of the variance in the predicted butyrate and myristic acid blood fluxes could be explained by these microbial species. *b*. visualizes the identified associations between the shown microbial species and the blood production potentials of the selected metabolites. The size of each stratum shows the number of connections for the microbial species (left stratum) and the selected metabolites (right stratum).

As the predicted alterations in blood metabolite productions are the result of the combined gut microbial alterations in each gut microbiome, single microbial species might not accurately explain the variation in the predicted fluxes (Figure 3). Therefore, we searched for microbial combinations that improved the correlation with the predicted fluxes over any single microbial species. Correlations were made for the combined relative abundances of all possible pairs, triplets, quartets, and quintets of microbial species with the predicted fluxes (Methods). To find the most efficient combination, i.e., the smallest microbial combination that best correlated with the predicted fluxes, we extracted the largest top microbial combination for the selected metabolites that improved the correlation strength with the associated fluxes by |ρ|>0.05 (Table S9). This analysis identified that combining the relative abundances of *B. ovatus* and *R. intestinalis* resulted in an optimal correlation with the predicted L-leucine and leucylleucine fluxes in blood (Figure 4(a)). The best correlating cluster for any three microbial species, which added *F. prausnitzii* to the cluster, only improved the correlation coefficient by 0.045. *R. intestinalis* has been previously^29^ found to have lower relative abundances in PD microbiomes compared to controls in the analyzed samples, while *B. ovatus*, as previously discussed, has been found before to have stable relative abundances between the two groups in the analyzed samples. This observation suggests that the lower predicted L-leucine and leucylleucine blood fluxes in PD patients are likely better explained by *R. intestinalis* than by *B. ovatus*. Nevertheless, the inclusion of *B. ovatus* in the top correlating microbial combination indicates that *B. ovatus* is potentially an important influencer of L-leucine and leucylleucine levels in blood, and that alterations in *B. ovatus* relative abundances likely influence gut microbial contributions to L-leucine and leucylleucine productions. When comparing this result to the previous microbial correlations with L-leucine and leucylleucine fluxes, it can be seen that both *R. intestinalis* and *B. ovatus* were not among the top correlates with L-leucine and leucylleucine blood fluxes (Figure 3(g)). The apparent synergistic effects of *R. intestinalis* and *B. ovatus* might be explained by complementary roles in the L-leucine pathway and independent microbial growth due to different ecological niches in the gut microbiome. This potential explanation was supported by a lack of association when correlating *R. intestinalis* and *B. ovatus* relative abundances (*p* = 0.58).

For butyrate production in blood, an optimal cluster of four microbial species was found consisting of *B. uniformis*, *F. prausnitzii*, *B. wexlerae*, and *E. rectale*. The combined relative abundances of these microbial species resulted in a Spearman correlation coefficient of 0.81 (Figure 4(a)), suggesting that these microbial species were the main microbial influencers of the predicted butyrate blood fluxes. Within this microbial cluster, *F. prausnitzii*, *B. wexlerae*, and *E. rectale* had lower relative abundances in the analyzed PD microbiomes, suggesting that these microbial species were likely the most important factors of the lower predicted butyrate blood fluxes in PD samples. This result further confirms *B. uniformis* as a major contributor to the predicted butyrate blood fluxes. Similarly to butyrate, a strong Spearman correlation was found for myristic acid blood fluxes and a microbial cluster consisting of *B. uniformis*, *B. vulgatus*, *F. prausnitzii*, and *Prevotella copri* (ρ = 0.77). As both *F. prausnitzii* and *P. copri* had lower relative abundances in PD microbiomes, these microbial species were likely major influencers of the lower predicted myristic acid fluxes. Next, for pantothenate, a cluster consisting of *Bacteroides dorei*, *B. vulgatus*, and *F. prausnitzii* was found. As *B. dorei* and *B. vulgatus* were not associated with PD microbiomes, the lower pantothenate fluxes were potentially influenced by *F. prausnitzii*. Finally, for nicotinic acid, the best correlating microbial combination contained *Alistipes putredinis*, *Collinsella aerofaciens*, *Phocaeicola plebeius*, and *R. lactatiformans*. Together, these microbial species negatively correlated with the predicted nicotinic acid fluxes (Figure 4(a)), indicating that a combined growth of these microbial species could contribute to lower nicotinic acid production in blood. Although the relative abundances of *A. putredinis*, *C. aerofaciens*, and *P. plebeius* were stable between PD microbiomes and controls, *R. lactatiformans* relative abundances were higher in PD microbiomes. This finding suggests that *R. lactatiformans* potentially contributed to the lower predicted nicotinic acid blood fluxes in PD patients, which aligns with the previously found association between *R. lactatiformans* relative abundances on predicted nicotinic acid blood fluxes (Figure 3(g)). Taken together, we identified combinations of gut microbial contributors to the predicted fluxes that best explained the flux predictions and found potential influencers of the predicted lower blood fluxes in PD patients. Overall, *F. prausnitzii* was most often found to explain the PD-associated predicted blood fluxes (Figure 4(b)), suggesting that this microbial species is a potential key microbe in gut microbiome influences on blood metabolism in PD.

## Discussion

For this study, we systematically profiled the potential metabolic contributions of gut microbiota to blood metabolites with replicated associations to PD diagnosis in a cross-sectional cohort of 654 personalized *in silico* models of host-microbiome co-metabolism. We also introduced a novel method for extracting species-specific microbial contribution potentials from the modeling predictions and identified gut microbial species that best explained predicted blood fluxes in the profiled *in silico* models. Together, our findings identified which blood metabolomic disturbances in PD may be linked to particular gut microbiota. Although it remains an open question whether gut microbial shifts are a causal factor of PD, as stated by Braak’s hypothesis,^48^ our results showed that gut microbial alterations in PD could exacerbate metabolic dysfunction in PD.

Our analysis identified six metabolites with lower gut microbiome contribution potentials to blood fluxes in PD microbiomes compared to controls, including L-leucine, leucylleucine, butyrate, nicotinic acid, pantothenate, and myristic acid (Figure 2). These modeling predictions were largely validated with previously meta-analyzed metabolomic studies that found diagnosis-related associations for these metabolites, suggesting a non-trivial role for the gut microbiome in these metabolomic associations with PD diagnosis. Only the lower predicted gut microbiome influences on blood myristic acid were not directionally linked with blood metabolomic findings in PD patients. Myristic acid is, however, commonly sourced from dietary intake of a wide variety of animal and plant products,^49^ so these discordances between blood metabolomic levels and predicted gut microbial contributions could be explained by dietary myristic sources. Moreover, lower microbial productions of myristic acid have also been observed in transgenic rhesus monkeys with induced neuronal alpha-synuclein aggregation.^50^ We further found inconsistencies between our predicted gut microbiome contributions and previous metabolomic findings of butyrate, L-leucine, and nicotinic acid levels in urine and plasma (Table 3), suggesting a disconnect between microbial influences of these metabolites and their blood levels. Previously, a similar disconnect between gut microbiome butyrate production and blood butyrate levels has been observed by Elford et al. (2024),^51^ who noted that butyrate is consistently decreased in stool samples, but not in blood samples. It has been proposed that this disconnect might be explained by increased leakage of microbial metabolites into the systemic circulation^39,52^ due to impaired gut barrier integrity in PD patients.^53^ Although the influence of dietary intake should not be overlooked, increased intestinal leakage in PD patients could also explain the discordance with previous metabolomic associations with butyrate, L-leucine, and nicotinic acid, as gut barrier integrity was not captured by the microbiome-personalized WBMs. We further found that the predicted flux associations with PD status were independent of age in the analyzed samples. This result is likely explained by confounding influences of age in both idiopathic PD incidence and gut microbiome compositions.^54,55^ We also did not find any dependence of sex on the observed associations between the flux predictions and PD status. Although the influence on sex in the gut-brain axis of PD patients remains understudied, estrogen, a regulator of the gut microbiome and microbially produced hormone,^56^ has been previously hypothesized to underlie sex differences in PD.^57^ Our results, however, suggest that this possible sex-specific association with the gut-brain axis in PD might not extend to the analyzed blood metabolites. To summarize, the predicted PD-associated gut microbiome contributions to blood metabolites were largely confirmed with previous metabolomic findings. The remaining inconsistencies could be potentially attributed to alterations of gut barrier integrity in PD patients that were not captured by the microbiome-personalized WBMs. Nevertheless, these findings show that gut microbiota could drive previously found blood metabolomic alterations in PD patients.

By employing the mechanistic nature of the constraint-based modeling framework, we also linked the predicted metabolites in blood with gut microbial species and identified the main microbial influencers of the predicted blood fluxes. We confirmed previously reported microbe-metabolite associations with PD diagnosis, such as the reduced relative abundances of butyrate producers in the genera *Faecalibacterium*, *Blautia*, *Eubacteria*, and *Roseburia*.^58–60^ Our results further confirmed previous associations in PD patients between the *Roseburia* genus and both fecal nicotinic acid and pantothenate levels, and a negative association between the *Bifidobacterium* and fecal pantothenate levels^15^ (Figure 3(g)). We also identified novel microbe-metabolite pairs that associate with PD diagnosis. Higher relative abundances of *R. lactatiformans* in PD microbiomes were linked with increased consumption of nicotinic acid, resulting in lower gut microbiome contributions to nicotinic acid in microbiome-personalized WBMs of PD patients. Another novel PD-associated microbe-metabolite potential link was found for *M. smithii* and L-leucine, as higher relative abundances of *M. smithii* in PD could be linked with lower gut microbiome productions of L-leucine through increased L-leucine consumption (Figure 3). The archaea, *M. smithii* has been previously associated with reduced gut motility, a common feature of PD,^61^ through the production of methane from hydrogen and carbon dioxide.^62^ We further linked lower myristic acid productions of *P. copri* to PD diagnosis (Figure 3), confirming previous positive associations between fecal myristic acid levels and *P. copri*,^63^ and previously found lower *P. copri* abundances with PD diagnosis.^59,64^ In summary, the metabolic interrogations of microbiome-personalized WBMs confirmed previously known microbe-metabolite associations in PD patients and identified potential new mechanistic links between PD-associated microbial species and metabolites.

Previous COBRA modeling efforts on PD microbiomes^9,10^ associated them with widespread alterations in sulfur metabolism. This study, however, could not find such signals. In part, this lack of results on sulfur metabolites could be attributed to the fact that most of these sulfur-associated metabolites have not been associated with PD diagnosis in a replicated manner in the consulted meta-analysis.^32^ However, no signal was found for the previously identified^9^ sulfur-associated metabolites that were analyzed in this study, being L-methionine, L-cysteine, L-serine, and L-aspartate (Table S5). Hertel et al. (2019) mechanistically linked their predicted alterations to intestinal sulfur metabolism to increased relative abundances of *A. muciniphila* and *B. wadsworthia* in PD microbiomes. However, neither microbial species was associated with PD status in this dataset (Table S8), potentially explaining the lack of sulfur-related signals in our results. In an initial analysis of our gut microbiome dataset, Wallen et al.^29^ noted that the missing association of *A. muciniphila* might be explained by geographic factors, as a previous multi-center study only found a significant association for *A. muciniphila* in samples from the north of the USA, but not in the south (Alabama, USA).^65^ The two previous PD microbiome community modeling studies identified PD-associations with *A. muciniphila* in stool samples from Germany^9^ and Luxembourg,^10^ which have climates that are more similar to northern US states than Birmingham, Alabama. Although it remains unknown if geographic factors play a role in the characteristics of the analyzed microbiome samples, we should be mindful of its possible implications, as gut microbiota are not only differentially enriched in different world regions,^66–68^ but geographic differences can also overlap with environmental^69,70^ and socioeconomic^71,72^ risk factors for PD. Taken together, the lack of overlap with previous metabolic modeling studies suggests diverse roles of gut microbiota in PD and hints at multiple microbial pathways that could independently contribute to PD progression.

There has been an emerging interest in potential therapies, which modulate PD progression through probiotic, prebiotic, and antibiotic targeting of gut microbes in PD patients.^73,74^ Early studies on microbial therapies have found potential improvements in constipation symptoms^75–77^ and both motor and non-motor symptoms.^78–82^ Still, the mechanisms, through which such therapies could modulate PD symptoms and progression, remain largely unknown. Our findings on potential microbial links with metabolic disturbances in PD might contribute to improved understanding of the molecular mechanisms behind these microbial therapies. Moreover, our results might be informative for the development of novel microbial therapies that target specific metabolic dysfunctions in PD.

## Strengths and limitations

The main strength of this study lies in the systematic integration of gut microbiome and metabolomic knowledge sources through personalization of WBM reconstructions and informing their analysis. This approach enabled the discovery of mechanistic hypotheses on microbe-metabolite links in PD patients. The gut microbiome data used for personalizing the microbiome-personalized WBMs was well-characterized and of high resolution as a result of using deep shotgun metagenomic sequencing. Moreover, the recently introduced APOLLO^25^ resource in combination with AGORA2^24^ enabled a high coverage of the original microbiome reads in the microbiome-personalized WBMs. Another strength was the rich sample metadata on lifestyle factors, disease comorbidities, medication usage, and technical variables. These metadata enabled our analysis to control for a broad range of confounding factors and reduced the risk of spurious findings. Despite this rich metadata, information on PD progression, cognitive decline, and dietary habits was missing. The inclusion of metadata on cognitive ratings, such as the Hoehn and Yahr scale,^83^ the Unified Parkinson’s Disease Rating Scale,^84^ and the Montreal Cognitive Assessment,^85^ could have helped with a more nuanced understanding of shifts in host-microbiome co-metabolism along PD progression. Moreover, information from food frequency questionnaires could have enabled the identification of links between dietary habits and gut microbial contributions to metabolic changes in PD. This study was also limited by the lack of blood metabolomic data to directly validate the modeling predictions. We tried to overcome this limitation by i) only investigating metabolites with replicated associations to PD diagnosis and ii) qualitatively validating the modeling predictions with previous human metabolomic findings from studies that passed quality assessment for a meta-analysis.^32^ Furthermore, the metabolic flux predictions did not capture intrinsic time-dependent dynamics of host-microbe and microbe–microbe interaction due to the fluxes being predicted in a network-wide metabolic steady state. The linear dependence between the predicted leucylleucine and L-leucine fluxes was likely an FBA-specific result, as linear dependence in metabolomic concentrations of leucylleucine and L-leucine concentrations might not be found due to differences in metabolic breakdown rates, molecular modifications, and cellular consumption and secretion rates. The models also did not capture relevant physiological features of the gut-brain axis in PD, such as changes in host-microbiome steroid hormone production^86^ and reduced intestinal barrier function.^53^ Future studies could mitigate these modeling limitations by combining WBMs with dynamic physiologically based pharmacokinetic models. Physiologically based pharmacokinetic models have been previously shown to successfully model the metabolic effects of oral estradiol intake^87^ and microbiome-mediated intestinal metabolic absorption.^88,89^ Moreover, the integration of WBM reconstructions with physiologically based pharmacokinetic models has already been demonstrated for modeling whole-body metabolic effects of ethanol and acetaldehyde exposure.^90^

## Conclusions

In conclusion, we identified blood metabolic markers of PD whose associations with PD diagnosis could be explained by compositional changes in the gut microbiome. In addition, we identified gut microbial species that may act as key drivers of these predicted gut microbiome influences. Our results included previously observed microbe – metabolite associations, such as *F. prausnitzii* with butyrate, but also included potential novel associations in PD, such as *M. smithii* and L-leucine. Importantly, the identified associations are likely not comprehensive and may reflect a small subset of all relevant microbe-metabolite interactions in PD. Moreover, experimental validation will be necessary to clarify the biological significance of the identified microbe-metabolite associations to PD disease progression. Taken together, this work linked gut microbiota with perturbed metabolites in PD patients. We hope our findings can contribute to the development of targeted microbiome-based interventions that modulate disrupted metabolic pathways in PD.

## Materials and methods

### Participant recruitment

Participant recruitment has been described before by Wallen et al.^29^ Briefly, participants were enrolled in the city of Birmingham, Alabama and the surrounding region between October 2018 and March 2020. The eligibility criteria for PD cases have been a confirmed PD diagnosis by a specialist at the Movement Disorder Clinic at the University of Birmingham, Alabama, and informed consent of the PD patient. Healthy controls were recruited from spouses or friends of the PD patients. Eligibility criteria for these controls were the absence of a PD diagnosis, and a lack of the following disorders: Alzheimer’s disease, dementia, REM sleep behavior disorder, multiple sclerosis, ataxia, dystonia, autism, epilepsy, stroke, amyotrophic lateral sclerosis, self-reported schizophrenia, and bipolar disorder. In addition, healthy controls were preferably aged 50 or older to ensure an age-matched cohort. Enrollment and sample collection was stopped in March 2020 to avoid confounding influences of COVID-19 and stress related to the COVID-19 pandemic. All sample processing steps, including DNA extraction, shotgun sequencing, quality and control steps, taxonomic profiling, and the collection of metadata, has been carried out blinded by Wallen et al.

### Retrieval of gut metagenomics and metadata

The gut metagenomics reads and the associated metadata were retrieved from a publicly available archive: https://zenodo.org/records/7246185. The retrieved gut metagenomics were already preprocessed and taxonomically profiled and have been comprehensively described here.^29^ Briefly, the metagenomics data were obtained from stool samples acquired from 490 PD patients and 234 neurologically healthy controls. The stool samples were sequenced by shotgun sequencing on an Illumina NovaSeq 6000, which included positive and negative controls. Human contamination has been removed by mapping the quality-controlled reads to the GRCh38.p13 human reference genome (https://www.ncbi.nlm.nih.gov/datasets/genome/GCF_000001405.39/). Taxonomic profiling has been performed with MetaPhlAn3,^91^ using the CHOCOPhlAn V30 metagenomic reference database (http://cmprod1.cibio.unitn.it/biobakery3/metaphlan_databases/). The participant metadata was previously obtained from questionnaires without interference from investigators (https://static-content.springer.com/esm/art%3A10.1038%2Fs41467-022-34667-x/MediaObjects/41467_2022_34667_MOESM1_ESM.pdf.)

### Metagenomic mapping

Microbial species in the retrieved gut metagenomics were mapped onto genome-scale metabolic reconstructions in the combined AGORA2^24^ and APOLLO^25^ resources, using the MARS pipeline.^92^ After mapping, the metagenomic reads were normalized by calculating the relative read abundances per sample. To limit false positives, sample relative abundances below the threshold of 0.00001% were further removed, whereafter the read abundances were normalized again. The mapping coverages, i.e., the fraction of available metagenomic data in a sample after mapping, were calculated from the original and mapped relative abundances after removing the reads below the relative abundance threshold.

### Microbial metabolic reconstructions

Genome-scale metabolic reconstructions for the mapped microbial species were retrieved from AGORA2 (version 2.01, https://www.vmh.life/files/reconstructions/AGORA2/.) and APOLLO^25^ (version 1.0 https://doi.org/10.7910/DVN/PIZCBI). As the metabolic reconstructions in AGORA2 and APOLLO are strain-resolved, pan-species models were generated to match the mapped species in the metagenomic data. The pan-species models were generated using the *createPanModels.m* CobraToolbox^18^ function and contained all unique reactions, metabolites, and genes in the set of AGORA2 and APOLLO strains within each microbial species in the mapped metagenomics data.

### Microbiome community models

Personalized metabolic models of gut microbiome communities were created using the mgPipe module in the Microbiome Modelling toolbox 2.0.^93^ The creation of microbiome community models has been described elsewhere.^94^ Briefly, pan-species models that mapped onto the gut metagenomics data in a sample were connected to a shared microbiota lumen, while transport reactions were added to enable metabolic exchanges between the pan-species models and the shared microbiota lumen. The mapped relative abundances were then integrated into the microbiome communities via the addition of a community biomass reaction (VMH reaction ID: communityBiomass), which lists each microbe’s biomass reaction with the microbe’s relative abundance as the stoichiometric coefficient. The community biomass reaction ensured proportional microbial growth for each integrated pan-species model per the mapped relative species abundances. Consequently, the community biomass reaction ensured that the metabolic contribution capacity of each microbial species to the host was proportional to their relative abundances.

### Whole-body metabolic models

We used generic sex-specific male and female WBMs^26^ (version 1.04c) for the host part of the microbiome-personalized WBMs in this study. These organ-resolved WBMs captured the metabolism of 26 organs and 13 biofluid compartments, which were connected in an anatomically accurate manner. The male generic WBM included 83,395 reactions, 58,095 metabolites, and 105,936 constraints, while the female generic WBM had 85,892 reactions 60,537 metabolites, and 109,757 constraints. All parameters were set to their defaults.

### Microbiome-personalized WBMs

The microbiome-personalized WBMs were created using the *combineHarveyMicrobiota.m* function, which joined the personalized microbiome community models with the large intestinal lumen compartment of the generic WBMs, as described previously.^26^ This function also added transport reactions to enable metabolic exchanges between the microbiota and the host. Moreover, this function updated the coupling constraints for each microbe’s reaction to their biomass reactions, to a value of 400, following previous WBM modeling studies.^26–28,95^

Further parameterization of the WBMs was done by setting the upper and lower flux bounds of the *Excretion_EX_microbiota_LI_biomass[fe]* reaction to one mmol/day/person. This reaction excretes the *microbiota_LI_biomass* compound, which is produced by the *communityBiomass* reaction. Fixing the flux bounds of the *Excretion_EX_microbiota_LI_biomass[fe]* reaction ensured that all generated WBMs produced the same metabolic flux over their microbiome community, despite differences in community compositions. In addition, the upper and lower flux bounds of the *Whole_body_objective_rxn* were set to one mmol/day/person, following previous WBM studies.^26–28,96^ The *Whole_body_objective_rxn* includes a linear combination of the whole-body organ biomass productions and associated coefficients for the proportional organ weights. This additional constraint ensured proportional organ biomass production between samples, corresponding to the organ weights.

### Diet

The microbiome-personalized WBMs were further parameterized with dietary uptake reactions and reaction constraints from the Virtual Metabolic Human database (https://www.vmh.life.).^31^ Reactions and constraints were chosen for the average European diet, as sufficient information on dietary uptake was not available. The dietary uptake formulation represented the metabolite-level average intake of a one-day meal plan for a 70 kg adult^31^ and was added to the models using the *setDietConstraints.m* CobraToolbox function. The added diet contained 192 metabolites (Table S3), including trace amounts, i.e., dietary uptakes below 0.1 mmol/day/person, of 92 metabolites.

### Simulations

The metabolic fluxes in blood were predicted by performing FBA, which translates a genome-scale metabolic reconstruction into a numerical model and interrogates the model using a linear programming solver according to an objective function.^33^ The objective function describes a linear optimization problem for finding extreme points in a constrained solution space of metabolic states as follows:

$$\underset{v}{\max}\;\;c^{T}\cdot v$$

(1) s.t,S⋅v=0,

lb≤v≤ub

The linear programming solver then finds a reaction flux vector *v* containing a maximized metabolic flux through an objective reaction, which is defined by a single non-zero coefficient in *c*. The maximized flux value must lie in the feasible solution space defined by constraints, such as the steady state constraint. Steady state is defined by the system of linear equations, S\cdotv=0, and enforces unchanging metabolite concentrations over time by ensuring that the amount of produced metabolites (products) equals the amount of consumed metabolites in each reaction (substrates). The metabolic products and substrates in each reaction are encoded by non-zero coefficients in the stoichiometric matrix, *S*. Substrates are represented by a negative coefficient, while reaction products are indicated by positive coefficients. In addition to the steady state constraint, each reaction in *v* was constrained by reaction-specific upper (*ub*) and lower (*lb*) bounds, derived from literature sources such as thermodynamic data and experimental measurements. If no reaction information was available, reactions were left unconstrained by setting *lb* and *ub* to arbitrary values of −1,000,000 and 1,000,000 mmol/person/day (see also Thiele et al.^26^ for details). For the objective reactions, we introduced demand reactions to the WBM blood compartments (VMH name: [bc]), for each of the analyzed blood metabolites. Demand reactions are unbalanced left-sided reactions (e.g., DM_leu_L[bc]: 1 leu_L[bc] → ∅ for L-leucine in blood), commonly used for predicting the maximal potential metabolite productions in a system. Demand reactions have been used in WBMs before to predict blood biomarkers of single gene defects^97^ and gut microbiome flux influences associated with risk markers of Alzheimer’s disease.^28^ Demand reactions were separately introduced for each FBA using the *addDemandReaction.m* CobraToolbox function.^18^ FBA was performed for each model by running *fba = optimizeWBModel(model)* with the *model.osenseStr = ‘max’* parameter to ensure maximization of the flux through the objective reaction. All default parameters were used for the linear optimizations. After performing FBA, the maximized fluxes were extracted from the *fba.f* solution field and rounded to six decimals to remove potential numerical artifacts that can appear in small numbers.

### Outlier removal

Outlier samples in the predicted fluxes were identified from a robust PCA on the z-transformed fluxes, performed with the alternating least squares algorithm. Potential outliers were identified by taking the Euclidean norm of the principal component scores for each sample, weighted by the explained variance of each principal component. This operation finds the average distance of a sample in a principal component to the center of the flux data. Values close to zero indicate samples for which average fluxes were predicted, while high values indicate potential outlier samples across the predicted fluxes. Together with a visual inspection of the first three PCA components, the ranked outlier scores were used to manually select outlier samples to be removed.

### Metadata descriptions

The Fisher exact test was used to quantify sample differences in PD patients and controls for the binary variables in the metadata. The *fishertest.m* Matlab function was used to calculate the Fisher-exact p-values, the associated odds ratios, and the 95% confidence intervals. For the numerical metadata, including age in years, BMI in kg/m^2^, and total read counts, differences between PD patients and controls were tested by performing the Wilcoxon rank sum test, using the *ranksum.m* Matlab function.

### Differential flux analysis

Logistic regressions were performed to associate the predicted blood flux values (predictor) with disease status (response, PD/Control). The flux predictions were log2 transformed and normalized via Z-transformation for the regressions. The following confounders were included in the regressions: age in years at stool collection (Z-transformed), sex (male/female), alcohol intake (yes/no), laxative usage (yes/no), antihistamine usage (yes/no), mood medication usage (yes/no), pain medication usage (yes/no), sleep-aid medication (yes/no). Finally, the total sequence count (log10-transformed and Z-transformed) was added to account for technical biases in sequencing and read processing.

The confounder selection was informed by a previous confounder analysis on the same gut metagenomics by Wallen et al.^29^ Their confounder analysis included a multivariable association study on both the available metadata and the processed gut metagenomics using the MaAsLin2 pipeline.^98^ We also followed the decision of Wallen et al. to not include covariates for gastrointestinal issues, such as constipation, digestion issues, and rapid weight loss or weight gain, as these features are intrinsic to the PD disease phenotype and would mask PD status if included. As previously noted by Wallen et al., older age and male sex are also intrinsic risk factors for PD. Nevertheless, these household covariates were included to estimate general associations with PD status that are also representative of women and younger PD patients. The influence of age and sex on the flux predictions was evaluated by performing a second set of logistic regressions on the predicted fluxes associated with PD status. Age-related influences were investigated by adding an interaction term for flux and age to the logistic regressions as predictors of interest. Sex differences were subsequently analyzed using logistic regressions with an interaction term for flux and sex as the predictor of interest. The multiple logistic regressions were performed with the *fitglm.m* Matlab function, using all standard parameters. 95% confidence intervals were obtained using the Wald method by running the *coefCI.m* Matlab function. Finally, the regression p-values were adjusted to account for multiple testing using the Benjamini–Hochberg procedure,^99^ by running the *mafdr.m* Matlab function with the ‘BHFDR’ option set to true.

### Modeling validation

The predicted PD-associated blood fluxes were systematically validated by qualitatively comparing the direction of the predicted associations with previous literature. Validation studies were selected by consulting only studies that were included in the same meta-analysis^32^ used for selecting the analyzed metabolites with replicated PD associations. All included studies for modeling validation passed quality assessments by having QUADOMICS^100^ scores of eight or higher and a Newcastle-Ottawa Scale^101^ score of seven or higher.

### Shadow price analysis

Microbial influencers of the predicted fluxes were identified by retrieving shadow prices in the FBA solutions. Shadow prices are a routinely used metric for identifying the value of a metabolite toward an objective function. More precisely, shadow prices for a metabolite *i*, $\pi_{i}$, represent the inverse ratio of an incremental relaxation of the steady state constraint for a metabolite *i*, $\delta b_{i}$, and the resulting maximal change in the maximized flux through the objective reaction, δZ,

(2) $$\pi_{i}=-\frac{\delta Z_{i}}{\delta b_{i}}$$

To identify the microbial species that could have contributed to the flux predictions, we extracted the shadow prices of their microbial biomass metabolites. Microbial biomass metabolites have the useful characteristic that they are coupled through all associated reactions within the microbial pan model via flux capacity (coupling) constraints,^95^ which enforce a minimal flux through the microbial reactions of 400 times the flux through the biomass reaction. A consequence of these biomass reaction couplings is that if a microbial species produces any metabolite in the pathway of the predicted metabolites in blood, a non-zero shadow price is found. Therefore, the shadow prices of the microbial biomass metabolites indicate which microbial species can contribute to the predicted fluxes. Practically, the shadow prices were automatically computed when performing FBA and were extracted from *fba.y* after performing *fba = optimizeWbmodel(model)*. Specifically, the shadow prices of the microbial pan biomass metabolites were obtained, e.g., *panBacteroides_uniformis_biomass[c]*. To get the number of microbial influencers for each optimized reaction and each microbial species, we counted the number of microbial species with non-zero shadow prices for their biomass metabolite, e.g., *panBacteroides_uniformis_biomass[c]*. Here, a non-zero pan-biomass shadow price means that increased or decreased biomass production, i.e., increased flux through the biomass reaction of a microbial species, would result in an altered flux prediction. A numerical tolerance for the shadow prices of 1e-6 was used to identify shadow price values of zero. Finally, the total number of microbial influencers for the investigated reactions was calculated by taking the union of microbial species with non-zero shadow prices across the cohort. Taken together, the shadow price values of pan biomass metabolites were used to identify which microbial species could influence the predicted fluxes through the contribution of microbial metabolites, including precursors of the analyzed metabolites in blood.

### Microbe contribution analysis

The microbial species contribution potential was defined as the greatest possible change in the predicted flux between an analyzed microbiome-personalized WBM ($\mathrm{mWB}M_{\mathrm{full}}$) and a hypothetical reduced microbiome-personalized WBM, where the microbe of interest was removed ($\mathrm{mWB}M_{\mathrm{reduced}}$). Crucially, we assumed that the microbial abundances in $\mathrm{mWB}M_{\mathrm{reduced}}$ are identical to $\mathrm{mWB}M_{\mathrm{full}}$, except for the removal of the microbial species of interest. This assumption enabled the prediction of how much each microbial species in the WBMs could contribute to the predicted blood fluxes in an ideal scenario. The simplest method for calculating the microbial species flux contribution potentials would be to iteratively predict the change in blood fluxes after removing each microbial species in all WBMs. However, as this approach would be prohibitively expensive to compute, we used an alternative computationally efficient approach that extracted the microbial species contribution potentials from the already calculated FBA solutions, thus eliminating the need for additional FBA predictions. As previously described, the shadow prices of microbial biomass metabolites in WBMs reflect the ratio of the maximal change in predicted flux, δZ, with a change in the availability of microbial biomass compound *i*, $\delta b_{i}$, in the WBM. Each microbial biomass compound in the WBMs is produced through a microbial biomass reaction. Thus, a breakage of the steady state of biomass metabolite $b_{i}$ necessitates a corresponding (steady state breaking) change in flux through the microbial biomass reaction. Conversely, a breakage of steady state by a change in flux through a microbial biomass reaction will result in increased availability of the microbial biomass metabolite. Note that the availability of the microbial biomass only increased in this scenario because the artificial removal of microbial biomass metabolites in the *communityBiomass* reaction was constant, which was the result of enforcing the excretion of the output of the *communityBiomass* reaction, i.e., the total gut microbiome biomass production, to one mmol/day/person. Another consequence of fixing the production and excretion of the *communityBiomass* compound was that the steady state flux values of each microbial biomass reaction were fixed, as the defined constant removal of microbial biomass metabolites necessitates an exact opposite production rate of microbial biomass metabolites. The consequence of this linear relationship between changing biomass availability and corresponding changing fluxes through a microbial biomass reaction was that the maximal change in blood fluxes could be calculated from the associated shadow price value, $\pi_{\mathrm{biomass},i}$, and the change in biomass reaction flux. $\delta v_{\mathrm{biomass},i}$:

(3) $$\delta Z_{i}=-\pi_{\mathrm{biomass},i}\cdot \delta v_{\mathrm{biomass},i}$$

Note that this formula is a reformulation of the modified shadow price formula with the change in biomass reaction flux replacing the change in metabolite availability, $\delta b_{i}$, in the original formula. Another change was the multiplication of the shadow price value by −1, to reflect that a steady state-breaking increase in microbial metabolic contributions to the flux predictions resulted in increased blood flux productions and vice versa. Now, the maximal change in blood fluxes when removing and reintroducing a microbial species of interest could be simulated by setting the microbial biomass reaction flux of $\mathrm{mWB}M_{\mathrm{reduced}}$ to zero and the biomass reaction flux of $\mathrm{mWB}M_{\mathrm{full}}$ to the microbial biomass reaction flux value in the corresponding FBA solution. As the chosen microbial biomass reaction flux in $\mathrm{mWB}M_{\mathrm{reduced}}$ was zero, the potential microbial flux contributions could be calculated by multiplying the shadow price of a microbial biomass metabolite by the flux through the microbial biomass production reaction. Taken together, by multiplying the shadow prices of biomass metabolites with their corresponding biomass reaction fluxes, microbial flux contribution potentials were predicted in a computationally efficient manner.

### Flux–microbe associations

To identify potentially relevant microbial flux contributors, the bootstrapped mean average flux contribution was calculated for all microbial flux contributions to the six selected metabolites. Bootstrapping was performed with replacement for 50,000 samples for all potential microbial flux contributions and for each of the selected metabolites. Then, the gut microbial species were ranked on their mean potential flux contributions to the blood production of each metabolite and microbial subsets were obtained by filtering on the top ranked microbial species that together had a potential flux contribution of 95% of the sum of all flux-associated gut microbial species for each of the six selected metabolites. The pruned lists of potential microbial contributors to the predicted fluxes were then correlated to their respective flux predictions by performing Spearman correlations. Potential microbial clusters that best explained the predicted fluxes were found on the pruned lists of flux-associated microbial species using a “brute-force” method of i) calculating the summed relative abundances of all possible pairs, triplets, quartets, and quintets of flux-associated microbial species, and 2) performing Spearman correlations for all microbial combinations against the associated flux predictions. Optimal sizes of microbial combinations were then selected by finding the largest top microbial combination that improved the correlation strength with the associated fluxes by |ρ|>0.05.

### Software

Modeling work, including the construction and analysis of the microbiome WBMs, was done in MATLAB 2020b and MATLAB 2024b (Mathworks^TM^). In addition, the parallel computing toolbox, statistics and machine learning toolbox, and the bioinformatics toolbox were utilized to generate and analyze microbiome WBMs. The generation and analysis of the microbiome-personalized WBMs were performed using functions from the COBRA toolbox v3,^18^ including the microbiome modeling^93^ and PSCM^93^ toolboxes. The IBM ILOG CPLEX 12.10 linear solver (IBM Inc.) was used for performing FBA on the investigated metabolites. All visualizations were carried out in R 4.4.2, using the tidyverse package.^102^

## Code availability

All scripts can be accessed at https://github.com/ThieleLab/CodeBase.

## Supplementary Material

Supplemental Material

Supplementary tables.xlsx

## Data Availability

The APOLLO and AGORA2 resources can be downloaded freely from Harvard Dataverse (https://doi.org/10.7910/DVN/JAXTWY). The generated microbiome-personalized WBMs and the associated raw fluxes can also be freely obtained from the Harvard Dataverse (https://doi.org/10.7910/DVN/PUTXL9).
