# Supplementary material for: Metabolic modeling links gut microbiota to metabolic markers of Parkinson’s disease
Source: Gut Microbes. 2025 Sep 25;17(1):2554195. doi: 10.1080/19490976.2025.2554195 (PMC12477873; doi:10.1080/19490976.2025.2554195)
Supplement: Supplemental Material [file KGMI_A_2554195_SM7620.pdf]

Supplementary figures

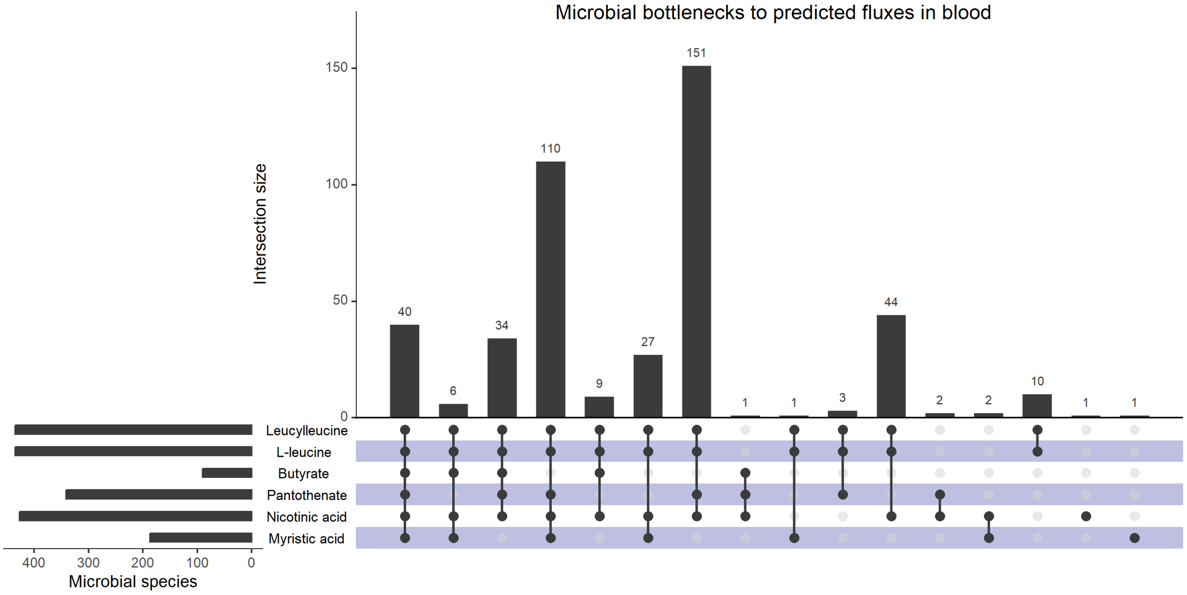

Figure S1: Number of microbial bottlenecks for predicted fluxes in blood in at least one sample. The Upset plot shows all non-empty intersections of microbial species that are associated with flux predictions of the six selected metabolites. The vertical bar plots show the number of species in each intersection of microbial sets. The associated metabolites of each intersection are indicated by the connected black circles under the vertical bars. The lack of unconnected black circles indicates that all microbial species were associated with two or more metabolites. The number of microbial species that are associated with predicted fluxes of each metabolite is shown by the horizontal bars on the left side of the figure. 40 microbial species were present in all metabolites (leftmost vertical bar). However, the largest intersecting set of microbial species was found for leucylleucine, L-leucine, pantothenate, and nicotinic acid, with 151 microbial species that were solely associated with these four metabolites.
